# Fusion of the word2vec word embedding model and cluster analysis for the communication of music intangible cultural heritage

**DOI:** 10.1038/s41598-023-49619-8

**Published:** 2023-12-20

**Authors:** Hui Ning, Zhenyu Chen

**Affiliations:** 1 The College of Humanities and Economic Management, Xian Traffic Engineering Institute, Xian, 710300 Shaanxi China; 2 The 20th Research Institute of China Electronics Technology Corporation, Xian, 710000 Shaanxi China

**Keywords:** Computational biology and bioinformatics, Engineering, Materials science, Mathematics and computing, Nanoscience and technology, Optics and photonics, Physics

## Abstract

This article aims to propose a method for computing the similarity between lengthy texts on intangible cultural heritage (ICH), to facilitate the swift and efficient acquisition of knowledge about it by the public and promote the dissemination and preservation of this culture. This proposed method builds on traditional text similarity techniques. The ultimate goal is to group together those lengthy texts on ICH that exhibit a high degree of similarity. First of all, the word2vec model is utilized to construct the feature word vector of music ICH communication. This includes the acquisition of long text data on music ICH, the word segmentation of music ICH communication based on the dictionary method in the field of ICH, and the creation of a word2vec model of music ICH communication. A clustering algorithm analyzes and categorizes ICH communication within the music. This procedure involves employing text semantic similarity, utilizing a similarity calculation method based on optimized Word Mover Distance (WMD), and designing long ICH communication clustering. The main objective of this analysis is to enhance the understanding and classification of the intricate nature of ICH within the musical realm. Finally, experiments are conducted to confirm the model’s effectiveness. The results show that: (1) the text word vector training based on the word2vec model is highly accurate; (2) with the increase in K value, the effect of each category of intangible word vector is improving; (3) the final F1-measure value of the clustering experiment based on the optimized WMD is 0.84. These findings affirm the usefulness and accuracy of the proposed methodology.

## Introduction

Intangible cultural heritage (ICH) is a subset of culture and a distinct body of knowledge that shares characteristics with other cultures. As a transmitter of ICH knowledge, the text has the advantages of a simple recording method and easy retrieval and can efficiently extract its fundamental content^1^. Because ICH knowledge encompasses a wide range of subject areas and various modes of expression, it must be expressed and managed in the textual form^2^. In most cases, it is impossible to efficiently organize related data since the texts comprising traditional intangible cultural assets lack linkage, leading to fragmented data. This lack of evidence falls short of people’s requirements, impeding the spread of information on ICH^3^. In addition, most domestic platforms for the application of ICH in vertical fields currently lack automatic association between non-textual data. Structured management of text data mostly relies on manual annotation, which undoubtedly incurs a high cost when attempting to establish associations between ICH textual data solely through this means.

However, with the advancement of natural language processing (NLP) technology, intelligent management of structured text has become possible. NLP is a vital component of Artificial Intelligence (AI). There is a strong focus on studying how to swiftly and efficiently extract and process meaningful information from texts and how computers comprehend and apply natural language^4^. The most fundamental activity in NLP is the determination of textual similarity. Both texts are vectorized using the proper technology, and then the degree of similarity between them is determined using an appropriate algorithm. The similarity between two texts is shown by the magnitude of the numerical number returned by the calculation^5^. Text similarity algorithms are at the heart of numerous applications, including the categorization and grouping of texts, AI-powered question answering systems, and search engines. The use of neural network technology in text similarity algorithms has grown in popularity in recent years as a result of an increased academic investigation of this area^6^. To give some examples, the Multilingual Unsupervised and Supervised Embedding (MUSE) model was originally suggested to express music emotions^7^. Then, a combined framework incorporating non-parametric fuzzy classification and evolutionary development was proposed for analyzing and producing music emotions^8^. Finally, the dominant masculinity depicted in Arab music videos was investigated, and the results revealed that it is predominantly characterized by traits of superiority, violence, and aggression^9^.

After conducting a thorough literature review, it was found that the word2vec model has a notable advantage in text clustering analysis and has made significant progress in this field. However, there is currently a limited amount of research on clustering long texts pertaining to ICH, and most studies have not accounted for the contribution of ICH terms. Furthermore, the traditional Word Mover Distance (WMD) distance only calculates weight based on word frequency and does not consider the contribution of ICH characteristic words, while also exhibiting issues with complexity and long calculation time when applied to long texts. To address these challenges, this article utilizes the word2vec model to construct feature word vectors for long texts related to music ICH, and subsequently applies clustering algorithms to perform cluster analysis on these texts. The article’s innovation lies in the enhancement of WMD, enabling the clustering of long texts related to ICH. The research objective is to establish a foundation for research and improvement of traditional text similarity methods, present a method for computing similarity between long ICH texts, cluster ICH texts with high similarity, and ultimately achieve efficient text management. This technique is anticipated to be employed in the future to construct knowledge graphs, facilitate full-text retrieval, and enable related recommendation functions on ICH platforms. As a result, the interconnections among various aspects of ICH knowledge can be effectively established, providing fast and efficient access to ICH knowledge for the general public. Furthermore, this will promote the diffusion and preservation of ICH culture.

## Research review

The word vector generating model has been the subject of extensive research. To address word2vec’s insensitivity to text word order information, Wang et al. introduced the Wang2Vec model. Wang2Vec is a model for generating word vectors that considers word order through a structured skip-gram model and a continuous window approach. Word order and semantic information are included in the resulting word vector^10^. Rui et al. proposed the Vector-Space Models for ambiguity. This technology saves word nuances by collecting data on their usage contexts^11^. The Sense2Vec model was proposed by Abdella and Uysal^12^. To produce word vectors to represent different word forms, the model based on the word2vec model performs part-of-speech tagging on the corpus during training. To give just one example, the word vectors produced contain both the adjective and adverb forms of the word^12^. Mahendra et al.^13^ used the Term Frequency-Inverse Document Frequency technique to find a middle ground between the advantages of the association between words and documents and words and corpus. They overcame the problem of existing word vectors not being able to effectively present a document’s data. It turned out that word2vec works well when combined with the estimated word weights^13^. Yadav et al.^14^ employed Convolutional Neural Networks (CNNs) in conjunction with attention mechanisms, leveraging deep learning techniques to develop a digital system for managing ICH and creating an automatic classification model. Furthermore, they discussed the fusion of knowledge graphs with deep learning to enhance knowledge management related to ICH^14^. Shen et al.^15^ introduced an approach centered on knowledge graphs, which established connections between elements of ICH and their cultural context and inheritance relationships. This facilitates improved preservation and exploration of ICH. They achieved a multimodal presentation and digital content management related to ICH by integrating text, image, and voice data with knowledge graphs^15^.

To sum up, the word2vec model has made some progress. However, there are not many studies on long text clustering of ICH, and most of them do not take into account the contribution of ICH. Based on these problems, this article discusses WMD improvement to achieve clustering of non-heritage long texts. The research content of this article mainly includes the word vector construction of music ICH communication based on word2vec and the clustering of music ICH long texts based on optimized WMD distance. The article aims to improve the dissemination and development of ICH.

## Construction of feature word vectors for music ICH communication based on word2vec

### Long text data acquisition for music ICH

To perform cluster analysis on texts concerning ICH in the domain of music, it is essential to acquire a dataset containing these texts. Consequently, this article utilizes web scraping techniques to procure textual data associated with ICH in music, thus establishing the foundational dataset for subsequent cluster analysis. The research employed web crawlers to gather data concerning the dissemination of ICH related to music, efficiently managing a substantial volume of webpage links through well-designed request methods. Additionally, due to its Python integration, comprehensiveness, and high scalability, the Scrapy framework was selected as the method for web data scraping in this investigation.

Scrapy is currently the most widely used web crawler framework. It employs Python, a highly integrated and flexible programming language, to complete its tasks. With the Scrapy framework, a web crawler can be developed swiftly, and the crawler based on this framework is highly scalable and robust. Therefore, this article designs a web crawler for ICH data (hereinafter referred to as the “ICH crawler”) based on the Scrapy framework. Table 1 lists the specific operation steps.

**Table 1:** Steps of the scrapy-based ICH crawler.

Step number	Specific operation
First step	Determine the rules between related Uniform Resource Locators (URLs) through the structure analysis. Splice and combine URLs of specified pages by writing logic codes
Second step	Add the spliced URL to the URL library for queuing and waiting for crawling
Third step	Analyze the crawled page results, and obtain data as required

**Figure 1:**
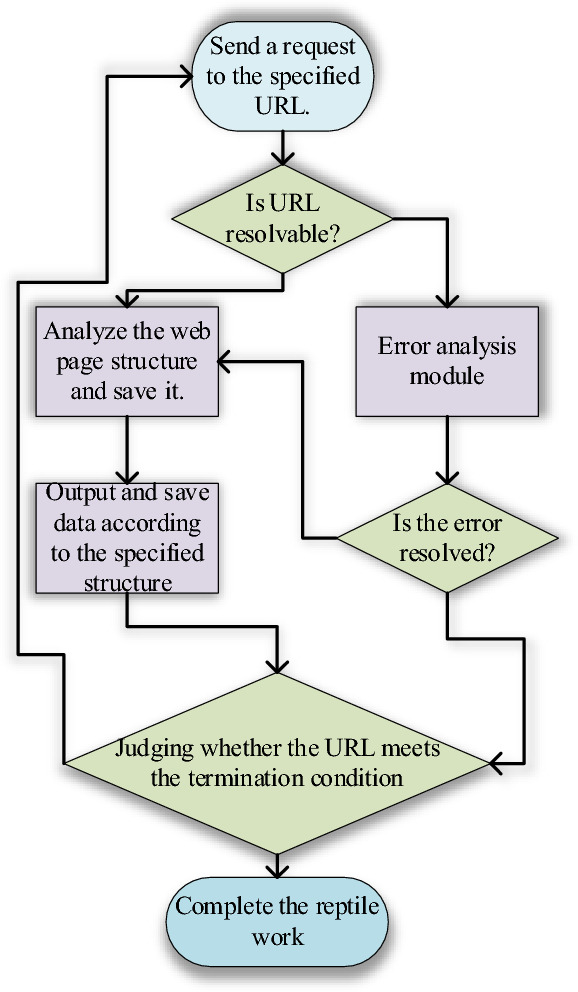
Design process of the ICH crawler.

Figure 1 depicts the primary process of the ICH crawler based on the aforementioned steps of the Scrapy data crawler.

Figure 1 depicts the main workflow of the data crawler designed here. It involves initiating a request to a designated URL, verifying if the URL can be parsed, analyzing and storing the webpage structure, outputting and saving the data as per the predefined structure, verifying if the URL meets the termination criterion, and ultimately concluding the crawling process.

### Word segmentation of music ICH communication based on the domain dictionary method

Efficient clustering analysis of text requires word segmentation to be performed. This section performs word segmentation on the dataset of ICH texts related to music, which has been constructed using a non-heritage domain lexicon method.

Textual information on the subject of ICH differs significantly from the ordinary textual information, particularly in the list of certain ICH items and some basic features, such as regions, scenes, acts, and attire. As a whole, the name of the ICH project needs to be distinguished. This section builds a lexicon in this area and combines it with the Jieba word segmentation tool to enhance the effect of Chinese word segmentation in the field of ICH.

Python’s Jieba word segmentation module combines dictionaries with statistical approaches for word segmentation to produce accurate results when processing Chinese text. First, the unique text is segmented using a trained Chinese prefix dictionary. Then, a directed acyclic graph is built for all possible situations that could constitute words in the text. Second, the maximum probability path is found using the dynamic programming method. Third, the maximum segmentation combination is determined using word frequency^16,17^.

In statistical word segmentation methods, Jieba effectively tackles the challenge of unregistered words in the text by leveraging the HMM. This is attributed to the HMM model’s exceptional performance in text segmentation, as it can recognize and segment unknown vocabulary by considering contextual information. Consequently, it adeptly manages text unique to specific domains. It calculates these unregistered words based on the Viterbi algorithm, and tags these words with parts of speech through the calculation results.

This article employs the ICH dataset related to music developed in the previous section to extract the list of music-related ICH projects from China’s National Intangible Cultural Heritage website. Subsequently, a lexicon for the ICH domain is generated using the data obtained. The Jieba method is applied to construct the lexicon, wherein each word was represented in a line with three components: the word, its frequency, and its part of speech. In this article, the word frequency is excluded, and a preliminary lexicon for the ICH domain is created in the format of “music category + specific quantity,” arranged alphabetically. The lexicon can be continuously updated and improved by monitoring and adding newly encountered unregistered words.

### Application of the word2vec model in music non-heritage texts

Furthermore, this article employs text representation to format natural language in a way that enables computers to more effectively analyze and compute it. Text vectorization, also known as word embedding, is a highly popular method for achieving this. There are two types of word embeddings, discrete and distributed. Of these, the distributed word embedding approach utilizing the word2vec model can map similar words to vector spaces in close proximity and frequently provides more precise semantic similarity representations. Consequently, this section employs the word2vec model to construct word vectors for a dataset of music ICH texts, thereby providing a solid groundwork for text clustering analysis. Moreover, the rationale behind choosing the word2vec model in this article is to harness text vectorization techniques, specifically distributional word embedding methods, to create word vectors that represent textual content associated with ICH in music.

#### Word2vec model

**Figure 2:**
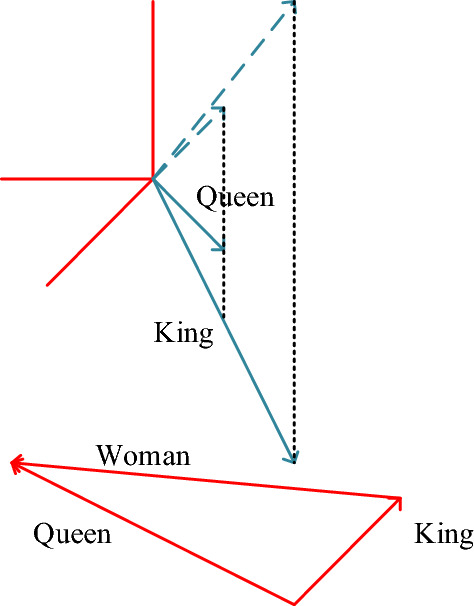
Relationship between word2vec word vectors.

The word2vec model was proposed by Mikolov et al. in 2013. The syntactic and semantic rules of the language are captured by the word vector formed using word2vec, and the semantic relationship between all words can be described by the vector’s offset^18^. Figure 2 displays a word2vec model as an example.

Figure 2 indicates that the word2vec model enables vector operations to be carried out between texts. Specifically, when the word vector for “King” is subtracted by the word vector for “Man” and added to the word vector for “Woman”, the result is approximately equal to the word vector for “Queen”.

The word2vec model is an improved neural network language model (NNLM). Table 2 lists the main changes and the advantages of these changes.

**Table 2:** Optimization and advantages of the word2vec model.

	Optimize content	Optimized effect
1	NNLM discards words that occur seldom	Word2vec now performs its calculations much more quickly and efficiently as a whole
2	NNLM tacks on the input layer’s word vectors	Word2vec reduces the amount of calculation of the model and improves the speed of training
3	NNLM context window size is randomized while removing hidden layers	Word2vec reduces the amount of calculation, and the training effect is not affected
4	The NLM training procedure employs the hierarchical soft-max technique and negative sampling technologies	The word2vec model has been effectively improved

One can choose between the Continuous Bag-of-Word (CBOW) model and the Skip-gram model in word2vec. The CBOW model estimates how often a determined word appears in the text by analyzing the frequency of occurrence of a set number of words before and after the location of W(t). In contrast to CBOW, Skip-gram uses the current word to forecast the probabilities of the two adjacent words. In the end, word2vec uses Hierarchical Softmax and NegativeSampling to train the model^19^.

#### Migration learning of the pre-trained word2vec model

Transfer learning is performed on a non-heritage text corpus based on the pre-trained word2vec model to guarantee the word vector effect. The basic tenet of transfer learning is to extract skills and knowledge from one area and apply them to another. If the transfer performance is high, we can save time and money on labeling data, significantly boosting the efficiency of our learning^20^.

This experiment presents the Chinese Wikipedia corpus for pre-training to produce the pre-trained word2vec model. This model ensures that the word vector has an accurate contextual relationship and mitigates the effect of insufficient collected data sets. It is important to check if the pre-trained model’s word vector dimension is the same as the new training’s word vector dimension. For this reason, the ICH corpus undergoes transfer learning to guarantee the coherence of the words.

#### Design of vector evaluation indicators for music intangible words

Evaluation of word vectors involves both introspective and objective measures. In the context of vector training technology, "internal evaluation" refers to the assessment of performance on individual intermediate subtasks. Simple and quick analogy subtasks, for instance, can aid in the comprehension of word vectors and allow for the quantitative evaluation of their efficacy. In most circumstances, it is not necessary to cover specific downstream jobs for evaluation. This evaluation approach is selected because a single NLP task can take a long time, and the effectiveness of word vectors will vary depending on the downstream task.

External parties ultimately evaluate word vector downstream tasks. Word vectors are only as efficient as the data they are trained on. After all, the word vector is the foundation of some NLP activities; therefore, some form of external evaluation is usually necessary. For this reason, internal review is still required to help pinpoint the source of poor downstream task model performance.

This article presents an experimental assessment for producing non-relic word vectors, which uses the correlation criterion. This criterion has the advantages of being rapid and straightforward to calculate.

First, K representative words with the characteristics of the ICH items are selected from the texts of each category of intangible cultural assets. For each word, the n most similar words are generated based on the cosine similarity of the intangible word vector, and the most pertinent words are selected based on subjective human judgement. The loss function is calculated by the cosine similarity ***Y*** between words and the subjective evaluation score ***f(x)*** (where the subjective evaluation value is determined to be the highest similarity value among n words). The evaluation index ***P*** is obtained according to Eq. (1).

1 $${\varvec{P}} = \frac{{\mathop \sum \nolimits_{1}^{{\varvec{K}}} {\varvec{L}}\left( {{\varvec{f}}({\varvec{x}}),\;{\varvec{Y}}} \right)}}{{\varvec{K}}}$$

In Eq. (1), ***K*** stands for the number of ICH representative words selected for each category; ${\varvec{f}}({\varvec{x}})$ represents the subjective score; ***Y*** signifies the cosine similarity between words; ***L*** refers to the loss function, which can be expressed as Eq. (2).

2 $${\varvec{L}}({\varvec{f}}({\varvec{x}}),\;{\varvec{Y}}) = \left| {{\varvec{Y}} - {\varvec{f}}({\varvec{x}})} \right|$$

Equation (2) indicates that the selected loss function in this model is the absolute value loss function. This function is obtained by computing the difference between the predicted value and the target value, and then taking the absolute value of the result.

The average evaluation value is calculated according to the evaluation criteria obtained for each category. After normalization processing, the final evaluation index is between 0 and 7. The higher the value, the more semantic information the word vector contains, and the better the effect of the training model.

## Long music ICH communication clustering based on optimized WMD

A strategy for developing feature word vectors was proposed in the previous section for large texts representing intangible cultural resources. Based on these vectors, the ICH long texts are grouped. The WMD method is used as a similarity calculation method for clustering non-heritage long texts. However, there are two issues that need to be addressed. First, the weight calculation method is overly simplistic. Second, the computation complexity is excessively high. This section refines these problems to effectively improve the contribution of ICH feature words in ICH communication and reduce the time complexity of the original method.

### Text semantic similarity

#### Text similarity based on the topic model

A topic model is a statistical model used in unsupervised learning to extract hidden semantic information from a corpus of text. Its primary application is to extract the latent topics from the corpus text and represent them in a dispersed manner to convey the text’s semantic content. In the topic model, each document consists of topics, and each topic is composed of topic-specific words.

Compared to the bag of words model, the topic model is more accurate and partially resolves the issue of erroneous results caused by ignoring the semantic relationship between words. However, the topic model has its limitations. For instance, the latent semantic model requires a considerable amount of time for singular matrix decomposition. When the training dataset is relatively large, this can lead to time-consuming complications^21^.

#### Text similarity based on WMD

The word2vec model is superior to the topic model in both accuracy and efficiency, as it can reflect the semantic relationship between words. Since its proposal, researchers have embraced this model due to its high performance in training on huge datasets.

WMD introduces word2vec into the Earth Move Distance model to calculate the similarity between texts. WMD trains the text based on the word2vec model to generate word vectors with semantic information. To determine how similar two texts are, WMD incorporates the word2vec algorithm into the Earth Move Distance model. WMD trains the text to generate word vectors that include semantic information using the word2vec model^22^.

### Similarity calculation method based on optimized WMD

The central concept behind the WMD algorithm is to evaluate the cost of moving all word pairs within a text, and subsequently, measure the similarity between texts. Nonetheless, the significance of words in a text is typically disparate, especially in the context of ICH texts used here where the importance of heritage-related terms outweighs other terms. To optimize the WMD distance, this article selects feature word vectors that accurately represent the ICH texts and applies enhanced weighting coefficients to replace the original WMD weights^23^.

Prior to performing text similarity calculations, the article initiates by defining text weights through the Term Frequency-Inverse Document Frequency (TF-IDF) method. The TF-IDF method, a widely adopted approach for calculating text feature weights, serves to assess the importance of individual words within a text. It expresses word significance as the product of two distinct components. Subsequently, a transition matrix denoted as T is constructed to represent the transition costs from keywords in one text, D, to keywords in another text, D’. The primary function of this matrix is to govern and assess the cost and importance of keyword transitions between texts.

The selection of the Euclidean distance as the metric for calculating transition costs between keywords is motivated by its ability to naturally measure the similarity or dissimilarity between keywords. This method incorporates the spatial distribution of keywords, providing a more realistic representation of their actual relationship. Utilizing the Euclidean distance in this manner contributes to a more accurate capture of the semantic associations between keywords, thereby enhancing the overall calculation results of text similarity.

There are two texts, ***d*** and ***d***′**,** in which a certain keyword of ***d*** is ${\varvec{k}}_{{\varvec{i}}}$*,* and that of ***d***′ is ${\varvec{k}}_{{\varvec{j}}}$*.* Here, the Euclidean distance is used to calculate the transfer cost between keywords of two documents, as shown in Eq. (3).

3 $${\varvec{dist}}({\varvec{k}}_{{\varvec{i}}} ,\;{\varvec{k}}_{{\varvec{j}}} ) = \sqrt {({\varvec{k}}_{{\varvec{i}}} - {\varvec{k}}_{{\varvec{j}}} )^{2} }$$

In Eq. (3), ${\varvec{dist}}({\varvec{k}}_{{\varvec{i}}} ,\;{\varvec{k}}_{{\varvec{j}}} )$ signifies the Euclidean distance between the key words ${\varvec{k}}_{{\varvec{i}}}$ and ${\varvec{k}}_{{\varvec{j}}}$.

Next, this article constructs a new keyword transition matrix denoted as **T**′ = {***k***_1_, ***k***_2_,…, ***k***_***n***_}, where ${\varvec{T}}_{{{\varvec{k}}_{{\varvec{i}}} ,{\varvec{k}}_{{\varvec{j}}} }} \ge 0$ represents the transition cost of keyword*** k***_***i***_ in document *d* to keyword ***k***_***j***_ in document *d*′. Subsequently, enhanced weight coefficients ${\varvec{W}}_{{{\varvec{i}}|{\varvec{D}}}}$ are introduced. To ensure that all keywords in document* d* are transferred to document *d*′, the total transition cost of keyword ***k***_***i***_ in document *d* is set to be equal to its corresponding enhanced weight coefficient ${\varvec{W}}_{{{\varvec{i}}|{\varvec{D}}}}$ (Eq. (4)). Similarly, the total cost of transitioning to *d*′ must also be equal to the respective enhanced weight coefficient of the corresponding keyword (Eq. (5)).

4 $$\mathop \sum \limits_{{{\varvec{j}} = 1}}^{{\varvec{m}}} {\varvec{T}}_{{{\varvec{k}}_{{\varvec{i}}} ,{\varvec{k}}_{{\varvec{j}}} }} = {\varvec{W}}_{{{\varvec{k}}_{{\varvec{i}}} |{\varvec{d}}}}$$

5 $$\mathop \sum \limits_{{{\varvec{i}} = 1}}^{{\varvec{m}}} {\varvec{T}}_{{{\varvec{k}}_{{\varvec{i}}} ,{\varvec{k}}_{{\varvec{j}}} }} = {\varvec{W}}_{{{\varvec{k}}_{{\varvec{j}}} |\user2{d^{\prime}}}}$$

Finally, the transfer cost ***C(d, d***′**)** of all keywords in text ***d*** to all keywords in text ***d***′ can be expressed as Eq. (6).

6 $${\varvec{C}}({\varvec{d}},\;\user2{d^{\prime}}) = \mathop \sum \limits_{{{\varvec{i}},{\varvec{j}} = 1}}^{{\varvec{m}}} {\varvec{T}}_{{{\varvec{k}}_{{\varvec{i}}} ,{\varvec{k}}_{{\varvec{j}}} }} {\varvec{f}}[{\varvec{dist}}({\varvec{k}}_{{\varvec{i}}} ,\;{\varvec{k}}_{{\varvec{j}}} )]$$

In Eq. (6), function *f* represents the mapping relationship from ${\varvec{T}}_{{{\varvec{k}}_{{\varvec{i}}} ,{\varvec{k}}_{{\varvec{j}}} }}$ to ${\varvec{dist}}({\varvec{k}}_{{\varvec{i}}} ,\;{\varvec{k}}_{{\varvec{j}}} )$. Based on the original WMD, the transfer cost is minimized using the new and enhanced WMD algorithm. When comparing two texts, the higher the transfer cost, the less comparable they will be. Thus, the final improved WMD algorithm can be written as Eq. (7).

7 $${\varvec{sim}}({\varvec{d}},\;\user2{d^{\prime}}) = \frac{1}{{1 + {\varvec{C}}({\varvec{d}},\;\user2{d^{\prime}})}}$$

In Eq. (7), to avoid calculates a transfer cost of 0, which would be smoothed and normalized by adding 1 to the denominator of the formula. To prevent the transfer cost of 0 from being calculated by ***C*** (***d***, ***d***′**)**, this article adds the denominator in Eq. (7) and normalizes and smooth it.

### Clustering design of long ICH communications based on optimized WMD

This article calculates the semantic similarity between non-heritage long texts based on optimized WMD, establishes their relevance, and clusters long ICH communication. This article also employs the K-means clustering algorithm to quickly cluster music ICH communication.

#### K-means clustering algorithm

The K-means technique is the most widely used approach to clustering. It is an unsupervised learning algorithm that relies on partitions to find patterns. When using the K-means clustering algorithm, ***n*** data points are partitioned into ***k*** groups, with each point being assigned to the group with the nearest mean (i.e., the cluster center). The criteria for grouping are governed by a set of rules^24,25^.

#### Clustering process of long music ICH communications

This article requires modifications to the standard K-means algorithm. These alterations mostly concern the following areas:

1. K = 5 is chosen for the clustering studies in this article because the ICH dataset used for the tests had five distinct categories.
2. In this experiment, one text object is selected as the initial clustering center in each of the five categories (folk songs, instrumental music, dance music, opera music, and Quyi music) of the music ICH dataset used for clustering experiments.
3. The clustering object of this experiment is the non-heritage text dataset, so the Euclidean distance used for calculation is replaced by the optimized WMD reported here.

**Figure 3:**
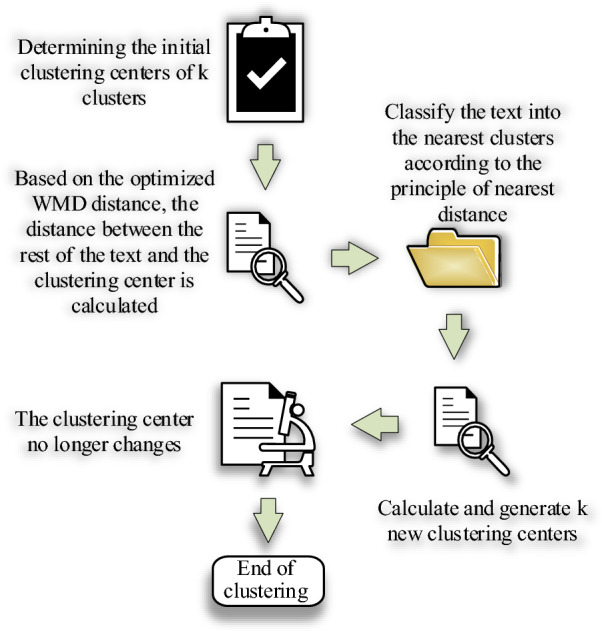
Improvement procedures of the K-means clustering algorithm.

Figure 3 presents the optimization process of the K-means algorithm.

### Experiment settings

#### Experimental data

The data used in this experiment was obtained through web crawling techniques from the collection of texts in the Chinese Intangible Cultural Heritage website. The data was divided into five categories: folk songs, musical instruments, dance music, opera music, and storytelling music. The obtained text results were then preprocessed, with a focus on filtering out texts with small data volumes. The specific contents of the data set required for the experiment are shown in Table 3.

**Table 3:** Distribution of experimental data.

Data category	Quantity	Average song size (Megabyte)	Language type	Average text length
Folk song	1200	1.5	Chinese	110
Instrumental music	1233	1.4	Chinese and English	96
Dance music	1201	1.6	English	103
Opera music	1134	2.1	Chinese and English	143
Quyi music	1156	1.5	Chinese	150

#### Experimental environment

Table 4 lists the hardware and software equipment parameters used in this experiment.

**Table 4:** Experimental environment parameters.

Environment type	Configuration parameters
Hardware environment	CPU: AMD Ry zen54600 H 3.0 GHz GPU: NVIDIA GeForce GTX 1650 4 GB Memory: 16 GB
Software environment	Operating system: Windows 10 Operating platform: Jupyter Notebook v6.0.3 Development language: Python 3.8.3

In addition, there are usually three types of evaluation indicators for clustering tasks: Precision, Recall, and F-Measure. This article uses these evaluation indicators to assess the experimental results.

#### Experimental content

Firstly, this article uses the NLP library genism in Python to train the constructed non-heritage music text dataset and evaluates the training results using evaluation metrics. Secondly, Jupyter Notebook platform is used to perform cluster analysis on the trained non-heritage music texts, achieving clustering of non-heritage music texts. Finally, a comparison is made between the clustering method based on WMD distance and the clustering method based on optimized WMD distance, to verify the effectiveness of the optimized clustering model.

## Model training results

### Training results of music ICH communication vectors based on word2vec

#### The training results of music ICH corpus word vectors

The word vector is trained using the word2vec module of Python’s NLP library, Gensim, and the corpus is trained using the transfer learning method. During the training, the dimension of the word vector in the pre-training model is guaranteed to be consistent with the dimension of the final word vector. Table 5 summarizes the results of training the word vectors.

**Table 5:** Comparison of training results of music ICH corpus word vectors.

Similar text	Folk song	Instrumental music	Dance music	Opera music	Quyi music
Similarity value	0.82	0.76	0.75	0.74	0.71

According to the data presented in Table 5, the word vectors of non-heritage music texts were trained and the resulting similarities for each category were found to be greater than 0.7. Specifically, the similarities for folk songs, musical instruments, dance music, opera music, and storytelling music were 0.82, 0.76, 0.75, 0.74, and 0.71, respectively. These results suggest that the word2vec model-based training of text word vectors is accurate and can lead to effective classification outcomes for text.

#### Evaluation results of ICH word vectors for music

**Figure 4:**
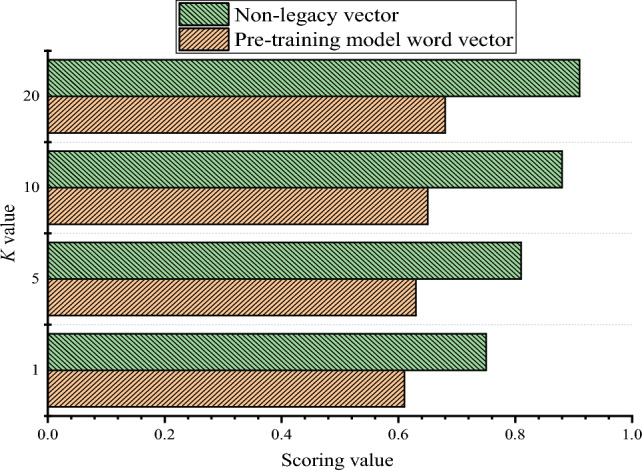
Evaluation index of the same word vector.

Figure 4 illustrates the evaluation results of word vectors via the pre-trained model and the intangible word vectors constructed here.

Based on the results shown in Fig. 4, the scores of the non-heritage word vectors for each category increase as the value of *K* increases. This suggests that a larger value of *K* can be less subject to subjective influence to some extent, but may require more time for manual annotation. Moreover, for the same *K* value, the evaluation indicators of the non-heritage word vectors are higher than those of the original training model, and contain more semantic information related to non-heritage. Thus, the effectiveness of constructing non-heritage word vectors has been validated.

### Results of clustering experiments on music ICH communications

#### Clustering experiment results based on optimized WMD

**Figure 5:**
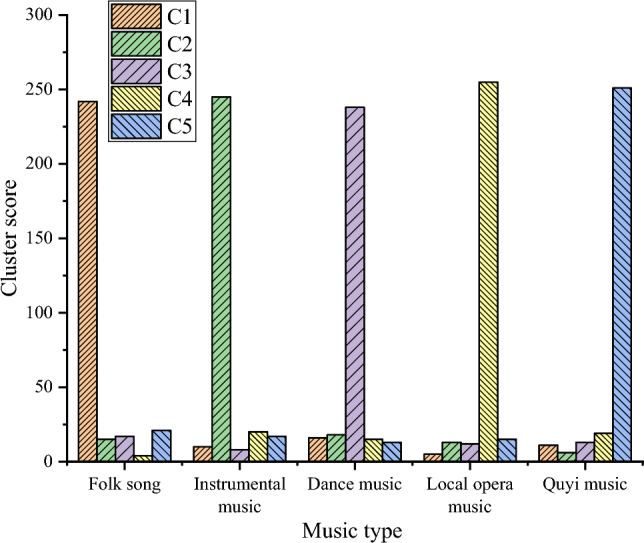
Experimental results based on optimized WMD clustering.

The optimized WMD is used to carry out the non-heritage long text clustering experiment after selecting the best *K* value. Finally, the experiment produced five clusters: C1, C2, C3, C4, and C5. Each cluster is described based on the category to which the majority of texts belong. The final result is that C1 is mapped to folk songs, C2 is mapped to instrumental music, C3 is mapped to dance music, C4 is mapped to opera music, and C 5 is mapped to Quyi music. Figure 5 reveals the clustering results.

Figure 5 shows that the F1-measure values for each category in the clustering experiment based on the optimized WMD are as follows: 0.83 for folk songs, 0.85 for instrumental music, 0.85 for dance music, 0.83 for opera music, and 0.84 for folk songs. The average of these values is used as the final evaluation metric. The final F1-measure value based on the optimized WMD for the clustering experiment is 0.84.

#### Evaluation of clustering results of different similarity methods

**Figure 6:**
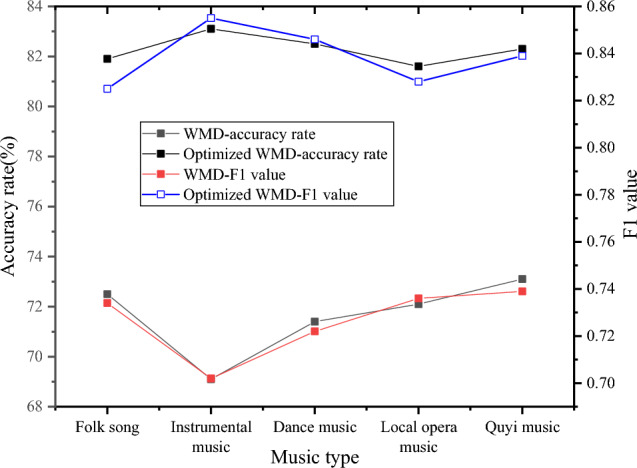
Different distance clustering experiment results.

This article compares the clustering method based on WMD and the clustering method based on optimized WMD, and Fig. 6 depicts the comparison results.

Figure 6 shows that the optimized WMD suggested here has a superior clustering effect for long ICH communications, outperforming the calculation technique based on WMD in terms of both accuracy and F1-measure value. The figure also demonstrates that text clustering using the WMD distance yields highly accurate results, indicating a significant level of similarity between the classified text and the clusters obtained during the training process.

Furthermore, to comprehensively demonstrate the effectiveness of the text clustering algorithm introduced in this article, it is evaluated alongside deep learning-based and knowledge graph-based text clustering algorithms previously proposed in the literature, respectively. In this case, a CNN is employed within the deep learning algorithm, featuring two convolutional layers and one fully connected layer. Each convolutional layer is comprised of 64 convolutional kernels with a kernel size of 3 and a pooling window size of 2. The model is trained using a learning rate of 0.001, a batch size of 32, and undergoes 20 iterations. A comparative analysis is conducted, and the outcomes of this comparison are presented in Table 6.

**Table 6:** Clustering performance of various algorithms.

Method	Accuracy (%)	F1-Score	Clustering Effect Description
Proposed optimized WMD method	85	0.88	Good
Convolutional neural network (Ref.^14^)	72	0.75	Moderate
Knowledge graph method (Ref.^15^)	62	0.60	Poor

Table 6 illustrates the performance comparison of various methods in clustering ICH texts. The proposed optimized WMD method stands out with a high accuracy of 85% and an F1-Score of 0.88, indicating excellent performance, particularly for clustering lengthy ICH texts. In contrast, while achieving moderate accuracy (72%) and an F1-Score of 0.75, the deep learning method slightly lags behind the optimized WMD method. On the other hand, the knowledge graph method displays poor performance, with both accuracy (62%) and F1-Score (0.60) falling below par. This result suggests that the knowledge graph method faces challenges in effectively handling ICH text clustering. Overall, the optimized WMD method excels in clustering ICH texts and holds promise for advancing the dissemination and development of ICH.

## Discussion

Due to issues of unclear segmentation features and low efficiency in current Chinese word segmentation methods, this article proposes a non-legacy word segmentation method based on a non-legacy dictionary. The method builds on the word2vec pre-training model to perform transfer learning on non-legacy language materials, laying the foundation for obtaining word vectors with non-legacy semantic information for subsequent experiments. Furthermore, this article proposes an optimized WMD distance based on the WMD distance and designs a new clustering method based on the optimized WMD distance. Experimental steps are constructed for non-legacy long-text clustering, along with evaluation criteria for clustering results based on word vector correlation. The effectiveness of the optimized WMD distance in long-text clustering is validated through experiments that analyze non-legacy long-text clustering using both WMD and optimized WMD distances, designed to verify the effectiveness of the optimized WMD distance in long-text clustering in different directions. Overall, as the category K value increases, the training effect of non-legacy word vectors for each category improves, which to some extent avoids subjective influences. However, when the number of experimental samples is too large, the efficiency of K-means clustering will significantly decrease. Future research will focus on developing clustering algorithms with better clustering effects. In summary, compared to traditional clustering methods, the clustering based on optimized WMD distance reported here exhibits higher precision and text matching similarity, making it more suitable for non-legacy long-text clustering. This task effectively manages non-legacy long-text by category, improving the correlation between non-legacy texts, and contributing to the inheritance and development of non-legacy culture.

## Conclusion

There are currently issues with low efficiency and accuracy in acquiring textual knowledge related to ICH. This article aims to improve the management and analysis of ICH texts for better cultural preservation by integrating the word2vec model into traditional text classification methods and combining it with the K-means clustering algorithm. The approach involves constructing word vectors and using cluster analysis to group music-related ICH texts, with the goal of enhancing the understanding and utilization of ICH knowledge. Experiments were conducted to verify the effectiveness of the proposed method, revealing that (1) the optimized WMD distance has a good effect on clustering music-related ICH texts, with an F1 value of 0.84; and (2) in the training of word vectors for music-related ICH texts, the similarity values for folk songs, instrumental music, dance music, opera music, and storytelling music are all above 0.7, indicating high accuracy of text vector training based on the word2vec model. However, a potential drawback of this article is that the processing efficiency of the model may decrease when the number of experimental samples is substantial. Therefore, additional clustering algorithms will be introduced for comparison purposes to identify the most effective algorithms for improving text clustering efficiency and facilitate the efficient management of ICH knowledge. These findings provide valuable insights for the development of text analysis methods.

### Supplementary Information

Supplementary Information.

## Data Availability

All data generated or analysed during this study are included in this published article [and its supplementary information files]. If someone wants to request the data from this study please contact the Corresponding author (Hui Ning, ninghui@xjy.edu.cn).
